# Juvenility and Vegetative Phase Transition in Tropical/Subtropical Tree Crops

**DOI:** 10.3389/fpls.2019.00729

**Published:** 2019-06-04

**Authors:** Muhammad Umair Ahsan, Alice Hayward, Vered Irihimovitch, Stephen Fletcher, Milos Tanurdzic, Alexander Pocock, Christine Anne Beveridge, Neena Mitter

**Affiliations:** ^1^ Queensland Alliance for Agriculture and Food Innovation, The University of Queensland, Brisbane, QLD, Australia; ^2^ The Volcani Center, Agricultural Research Organization, Institute of Plant Sciences, Rishon LeZion, Israel; ^3^ School of Biological Sciences, The University of Queensland, Brisbane, QLD, Australia

**Keywords:** miR156, miR172, miR156 targeted *SPLs*, phase transition, juvenility and maturity, flowering, *APETALA 1* (*AP1*), microRNA

## Abstract

In plants, juvenile to adult phase transition is regulated by the sequential activity of two microRNAs: miR156 and miR172. A decline in miR156 and increase in miR172 abundance is associated with phase transition. There is very limited information on phase transition in economically important horticultural tree crops, which have a significantly long vegetative phase affecting fruit bearing. Here, we profiled various molecular cues known to be involved in phase transition and flowering, including the microRNAs miR156 and miR172, in three horticultural tree crops: avocado (*Persea americana*), mango (*Mangifera indica*), and macadamia (*Macadamia integrifolia*). We observed that miR156 expression decreases as these trees age and can potentially be used as a juvenility marker. Consistent with findings in annual plants, we also observed conserved regulation of the miR156-*SPL3/4/5* regulatory module in these genetically distant tree crops, suggesting that this pathway may play a highly conserved role in vegetative identity. Meanwhile, the abundance of miR172 and its target *AP2-like* genes as well as the accumulation level of *SPL9* transcripts were not related with plant age in these crops except in avocado where miR172 expression increased steadily. Finally, we demonstrate that various floral genes, including *AP1* and *SOC1* were upregulated in the reproductive phase and can be used as potential markers for the reproductive phase transition. Overall, this study provides an insight into the molecular associations of juvenility and phase transition in horticultural trees where crop breeding and improvement are encumbered by long juvenile phases.

## Introduction

The plant life cycle can be divided into three distinct growth phases – juvenile, adult, and reproductive – all of which involve temporal and spatially coordinated changes in various traits essential for plant survival and reproduction. These phases may be thought of as developmental phases, with continuous development of new organs that possess different morphological features in each phase. Internode length, leaf length and size, trichome distribution, and cell size may vary in some species according to the developmental phase (Poethig, 2003; Huijser and Schmid, 2011; Wang et al., 2011). In *Arabidopsis thaliana*, early rosette leaves show juvenile features, smooth margins, small and almost round blades and long petioles. Meanwhile, late rosette leaves exhibit adult traits having shorter petioles, serrated margins, and abaxial trichomes (Telfer et al., 1997; Huijser and Schmid, 2011; Tsukaya, 2013). Heteroblastic features are also observed in maize (*Zea mays*), where adult leaves have trichomes but lack epicuticular wax, while juvenile leaves exhibit the opposite phenotype (Poethig, 2003). Likewise, differences in juvenile and adult leaf morphology can be detected in some woody species including *Acacia confuse, Acacia colie, Eucalyptus globulus*, *Quercus acutissima*, and *Hedera helix* (Wang et al., 2011).

Phase change transitions, associated with phenotypic changes in leaves, are known to be moderated by the sequential action of two main microRNAs: miR156 and miR172 (Poethig, 2003; Wu et al., 2009; Wang et al., 2011; He et al., 2018). microRNAs are small noncoding RNA molecules (20 to 24 nt) that negatively regulate eukaryotic gene activity posttranscriptionally. miR156 binds and target transcripts of *SQUAMOSA PROMOTER BINDING PROTEIN-LIKE (SPL) transcription factors* (*TFs*) for degradation. miR156 is highly abundant in the juvenile phase and decreases as the plant ages, whereas miR172, a repressor of *APETALA2 (AP2)-like TFs* has the inverse transcript abundance (Wu et al., 2009). In a broad sense, as miR156 levels start declining, the level of its targeted *SPL* genes starts increasing. These, in turn, upregulate the transcription of miR172, resulting in *AP2* TF repression, a condition that marks the transition from juvenile to adult phase.

Specifically, in *Arabidopsis*, miR156 targets 10 out of 16 *SPL* family members (*SPL2, SPL3, SPL4, SPL5, SPL6, SPL9, SPL10, SPL11, SPL13, SPL15*), all characterized by a 76-amino acid DNA-binding domain termed *SBP* (*SQUAMOSA PROMOTER BINDING PROTEIN*) (Birkenbihl et al., 2005; He et al., 2018). Whereas, on the other hand, miR172 targets six *AP2-like* transcriptional repressors including *APETALA2 (AP2), TARGET OF EARLY ACTIVATION TAGGED (EAT) 1 (TOE1), TOE2, TOE3, SCHLAFMUTZE (SMZ)*, and *SCHNARCHZAPFEN (SNZ)* (Aukerman and Sakai, 2003; Chen, 2004; Mathieu et al., 2009; Jung et al., 2014). Not surprisingly, studies performed in *Arabidopsis* revealed that overexpression of miR156 results in a long juvenile phase, whereas low levels of miR156 results in an early flowering phenotype (Schwab et al., 2005; Wang et al., 2009). As plants start maturing, the level of miR156 begins decreasing, a condition that allows production of *SPL9* and *SPL10* proteins (Huijser and Schmid, 2011). These *SPL* genes can bind to the sequences in the regulatory region of *MIR172b*, thus positively regulating miR172 transcription. The expression pattern of *MIR172b* is positively correlated with the adult phenotype (leaf epidermal traits) (Wu et al., 2009). Double mutants for *spl9/spl15* exhibit a late-flowering phenotype (Schwarz et al., 2008; Wu et al., 2009), while miR156-insensitive *SPL9 (rSPL9)* transgenic plants show early flowering and a high abundance of miR172 (Wu et al., 2009).

This increase in *SPL* and miR172 levels and subsequent repression of *AP2-like TFs* makes a favorable inductive condition to activate various meristem identity genes including *APETALA1* (*AP1*) (Wigge et al., 2005; Jung et al., 2016). *AP1* plays a vital role in promoting phase transition and also forms a central core with other flowering genes in the regulatory network for floral organ development in the meristem (Murai et al., 2003; Kaufmann et al., 2010; Chuang et al., 2018). Overexpression of *AP1* in birch results in dwarf plants with a shortened juvenile period and an early flowering phenotype (Huang et al., 2014). Furthermore, *Arabidopsis ap1* mutants show abnormal floral development and a late flowering phenotype (Ondar et al., 2008; Qi et al., 2011). *AP1* is known to be upregulated directly by *SPL3/4/5* which binds to the *AP1* promoter region (Wang et al., 2009; Yamaguchi et al., 2009; Jung et al., 2016). Moreover, *SUPPRESSOR OF OVEREXPRESSION OF CONSTANS 1 (SOC1)*, a key floral integrator, positively regulates *SPL3/4/5* activity, thereby facilitating activation of various floral meristem genes (Jung et al., 2012).

Alongside the main role that miR156 and miR172 play in controlling juvenile to adult and reproductive phase transition, it has been shown that other microRNA family members also take part in controlling flowering time. For instance, it has been recently documented in *Arabidopsis* that miR159 promotes vegetative to reproductive phase transition by targeting *MYB33*, which encodes an R2R3 MYB domain TF (Guo et al., 2017). Usually, *MYB33* promotes miR156 transcription, thus high miR159 abundance prevents over activation of *MIR156A* and *MIR156C*, keeping a delicate balance of phase transition events.

In addition to the miR159-*MYB* regulation of miR156, phase transition is thought to be regulated through as yet unknown signal(s) derived from leaves that also represses miR156 (Yang et al., 2011). Removing leaves from *Arabidopsis* and *Nicotiana benthamiana* plants resulted in an increased level of miR156 leading to a long juvenile phase. One potential candidate for this mobile repressor is sugar (Yang et al., 2013; Yu et al., 2013). Indeed recently, it was suggested that *Trehalose-6-Phosphate Synthase 1* (*TPS1*), a key enzyme in the T6P pathway which regulates carbohydrate availability, is essential for flowering in plants (Wahl et al., 2013). Specifically, in the *Arabidopsis* shoot apical meristem, sucrose and T6P are suggested to control flowering by inducing *SPL3/4/5 expression* either in a dependent or independent miR156 pathway. Accordingly, the *tps1* loss of function mutation causes extreme delays in flowering and reduction of *SPL3* transcripts (Wahl et al., 2013). This observed loss of function mutation was rescued during embryogenesis using an inducible *TPS1*; without this inducible transgene, the observed mutation was embryo lethal. To date, the involvement of the miR156-*SPL* and miR172-*AP2* regulatory modules, as well as other floral integrators in vegetative phase transition, has been shown to be conserved in annual, as well as in some perennial trees (Wu et al., 2009; Wang et al., 2011). However, it is still unknown whether the same mechanism controls phase transition in the vast majority of horticultural tree crops that are commercially significant for world food production. This information is critical as a long juvenile phase in horticultural trees poses a serious hindrance to crop selection and improvement because breeding and productivity traits are dependent on attaining reproductive status (i.e., flowering and fruiting). With this in mind, we examined the conservation of these regulatory modules in three important fruit tree species from diverse origins including: *Mangifera indica* (mango), *Persea americana* (avocado), and *Macadamia integrifolia* (macadamia). In each of these species, no visible heteroblastic changes can visually delimit vegetative phase transition, except presence of floral structures on a reproductively mature tree. However, each of these species experiences a significantly long juvenile phase affecting breeding and productivity. Avocado and macadamia possess a significantly long juvenile phase of 6–10 years, while mango has 3–5 years, depending on cultivar (Lavi et al., 1992; Mukherjee and Litz, 2009; Alam et al., 2018). In view of the absence of morphological markers during vegetative stages, we used plants of known ages and flowering competency to screen candidate miRNAs/genes from other model species and identify molecular markers to differentiate between a juvenile tree and a reproductively mature tree. Here, we show that miR156 transcript level in leaves correlates with juvenility and phase transition in these crops. We also show that miR156 expression anticorrelates with *SPL4* accumulation and other floral homeotic genes including *AP1* and *SOC1*.

## Materials and Methods

### Gene Identification, Sequence Alignment, and Phylogenetic Analysis

In-house transcriptomic and genomics resources were utilized for gene transcript identifications. For macadamia, recently published genomics resource was also utilized^1^. In addition to that, NCBI database^2^ was also searched to find already published gene transcripts (*PaAP1* and *MiAP1*) (Nakagawa et al., 2012; Ziv et al., 2014). BLAST searches were made for *Arabidopsis* homologs to the transcriptomic data to identify related transcripts using Geneious software version 11 (Supplementary Table S1; Kearse et al., 2012). These transcripts were then translated in all six frames. These translated frames were then aligned to the *Arabidopsis* transcripts to identify the correct frame and then further verified the integrity of “respective” protein domain through Pfam^3^ (Supplementary Table S1). These identified transcripts (CDS and translated frames) were then subjected to reciprocal blasts *via* NCBI and TAIR *Arabidopsis* online databases for further confirmation. Finally, psRNATarget^4^ online tool was utilized for miRNA target site prediction in respective miRNA target transcripts. Full-length CDS/Open Reading Frames (ORFs) were predicted manually by aligning and comparing all similar transcripts from different transcriptomic resources and further aligning to the genomic data. These ORFs then were further verified using ORF finder^5^ online tool for ORF prediction.

Multiple sequence alignment was completed using Geneious software default Muscle alignment setting (Kearse et al., 2012). To illustrate divergence of miR156-targeted *SPL* and miR172-targeted *AP2* transcripts, a genetic tree was constructed and visualized in MEGA 7 using Maximum Likelihood phylogenetic analysis method based on the JTT with Freqs. (+F) model from transcripts from avocado, mango, macadamia, and other plant species (Supplementary Tables S2, S3; Kumar et al., 2016).

### Tissue Collection and Grinding

Samples from 1 month to 2 years, youngest fully expanded leaf arising from the primary unbranched shoot was collected. For mature/already flowering trees planted in field, the general sampling strategy was to collect youngest fully expanded leaves from 15 different positions on each of three replicate trees (three biological replicates) to capture an average for each tree regardless of potential leaf-specific variation. Care was taken during sampling to source youngest fully expanded leaves of similar age/stage to avoid any variation due to the age of the leaves. The sampling age for mature/already flowering avocado and macadamia was chosen as 10-year-old field-grown trees since avocado and macadamia have a 7- to 10-year juvenile phase. On the other hand, mango cultivar Kensington Pride starts flowering in about 3–5 years, hence 5-year-old trees were selected as mature already flowering samples.

More specifically, the Avocado cv. ‘Hass’ seeds were provided by Anderson Horticulture Pty Ltd. and grown in Anderson nursery located in Duranbah, New South Wales, Australia. Fully emerged new leaves from 15 individual plants pooled into three groups/samples were sampled 1 month, 3 months, and 1.5 year after germination. Six mature/already flowering trees (approximately 10-year-old grafted on Velvick rootstock) were sampled during the time of flowering (spring) located at Maroochy Research Facility (MRF) Nambour, Queensland, Australia. Mango cv. Kensington Prides seeds were sourced from the fruit markets and grown in UQ23 soil in the University of Queensland Australia glasshouse. A total of 20 plants were sampled for 1 month, 4 months, and 1 year after germination. The samples for 5-year-old mango trees (six mature/already flowering true seedling trees) were collected from Donovan Family Investments farm in Bundaberg area of Queensland, Australia. Macadamia nuts for cv. HAES 741 were kindly provided by Macadamia Breeding team at MRF Australia and were grown in UQ23 soil at the University of Queensland Australia glasshouse. A total of 15 plants were sampled for 1 month and 6 months after germination. Six mature/already flowering macadamia trees were sampled during the time of flowering (spring) located at MRF. To avoid issues with circadian rhythms, all the samples were taken at same time of the day (10 am AEST). All the samples were taken during flowering seasons to avoid any possible seasonal variations. Samples were placed on dry ice immediately after sampling and cryogenically ground to a fine powder using a mortar and pestle in liquid nitrogen.

### RNA Extraction and cDNA Synthesis

Total RNA of collected samples was extracted using a MasterPure Plant RNA Purification kit (Epicentre, Madison, WI, USA) according to the manufacturer’s protocol (same biological samples were used both for small RNA sequencing and qRT-PCR). Five-hundred nanograms of high-quality total RNA were then further used for low-molecular weight cDNA synthesis (to quantify mature miRNAs) using a miScript Plant RT Kit (Qiagen, Venlo, The Netherlands) as per manufacturer’s protocol. For gene quantification, cDNA was synthesized using SensiFAST™ cDNA Synthesis Kit (Bioline, London, UK) on 600-ng total RNA as per manufacturer’s protocol.

### Small RNA Sequencing

NEXTflex Small RNA-Seq Kit v3 was utilized to prepare small RNA libraries per manufacturer’s instructions (Bioo Scientific Corporation, Austin, TX, USA). Briefly, 1 μg of total RNA extracted using the above method from 1-month and 10-year-old (5-year-old mango) avocado, macadamia, and mango tree leaves were utilzed. During 3′ adapter ligation step, the samples were incubated at 20°C overnight. Then, first strand cDNA was synthesized using NEB #M0253 kit according to manufacturer’s protocol using the NEXTflex RT Primer (New England Biolabs Inc., Beverly, MA, USA). Incubation at 25°C for 10 min was followed by extension at 37°C for 40 min and inactivation of the enzyme at 65°C for 20 min. NEXTflex reagents and primers were used for 17 cycles of PCR amplification. PCR products between 130 and 180 bp were cut from a 3% MetaPhor Agarose gel. The QIAquick gel extraction kit (QIAGEN, Venlo, The Netherlands) was used to isolate this DNA before libraries were sent to the Queensland University of Technology Australia, genomics laboratory for quantification and sequencing on Illumina TruSeq (Illumina, San Diego, CA, USA) as a 76-cycle single read library.

Raw read files were processed with Trim Galore^6^ to remove adapters, with trimmed reads of lengths less than 30 nt retained. Reads mapping to ribosomal RNAs were removed using BBduk^7^. The SCRAM pipeline was used for quantifying miRNA reads (Fletcher et al., 2018). Normalization of read counts was based on reads per million reads between 20 and 24 nt. Reference mature *Arabidopsis* and rice miRNA sequences for mapping were obtained from miRbase^8^.

To validate miR156, miR172, and miR159 conservation in these tree crops, the mature *Arabidopsis* sequence of known miRNAs (miRBase) was blasted against the above prepared small RNA libraries and were further aligned to verify 100% homology using Geneious software ver 11 (Kearse et al., 2012).

### Quantitative Real-Time Polymerase Chain Reaction

For miRNA quantification (cDNA prepared using a miScript Plant RT Kit was utilized), three biological replicates were utilized in duplicate for qRT-PCR using miScript SYBR® Green PCR Kit as per manufacturer’s instruction. Mature sequence of miRNAs was used as forward primer, and universal reverse primer was provided with SYBR kit (Supplementary Table S4). The qRT-PCR run was performed using a Rotor-Gene Q 6000 and was further analyzed using Rotor-Gene Q 2.3.1.49 software provided by the manufacturer (Qiagen, Venlo, The Netherlands).

For gene transcript quantification, *P. americana, M. indica,* and *M. integrifolia* gene specific primers were designed using Geneious Software (Supplementary Table S5). Transcript abundance was monitored in real time using SensiFAST™ SYBR® No-ROX Kit (Bioline, London, UK) on CFX384 Touch™ Real-Time PCR Detection System (Bio-Rad, Hercules, CA, USA) according to manufacturer’s instruction. This then was analyzed on CFX Manager™ Software provided by the manufacturer.

Transcript relative abundance was calculated based on primer efficiencies calculated using LinRegPCR 7.5 software (University of Amsterdam, Netherlands) (Ramakers et al., 2003) using the comparative cycle threshold (CT) method as described by Hayward et al. (2009). For statistical analysis, a one-way analysis of variance (ANOVA) was performed with *post hoc* multiple comparison tests using Tukey’s HSD correction (SPSS 23, IBM, USA). For gene transcript quantification, avocado and macadamia orthologs of both *GAPDH* and *EF1a* were used as housekeeping genes to compute relative expression. For mango transcript expression analysis, *MiGAPDH, MiEF1a, MiMON1,* and *MiUBQ10* were used to normalize transcript abundance. Averages of relative expression of each miRNA against the housekeeping gene U6 (Cheah et al., 2015), 5.8S rRNA (Chen et al., 2015b) were plotted with standard error using GraphPad Prism 6 (GraphPad Software Inc.).

## Results

### Monitoring the Expression Levels of miR156 and miR172 at Juvenile and Adult Stages

miR156 and miR172 are conserved plant miRNA families and master regulators for both juvenile to adult and adult to reproductive phase transition in plants, with each showing anticorrelating expression patterns in leaves over the plant life cycle (Schwab et al., 2005; Wu and Poethig, 2006; Gandikota et al., 2007; Wu et al., 2009; Jung et al., 2011; Wang et al., 2011; Zhang et al., 2015; Feng et al., 2016; Xu et al., 2016). To explore whether the transcript abundance (from leaf samples) of these two miRNAs changes with vegetative phase transition in horticultural tree crops, we confirmed their presence in mango, macadamia, and avocado using small RNA sequencing data (see Material and Methods). We then determined their expression levels by quantitative real-time PCR (qRT-PCR) in fully expanded leaves of seedlings over time, as well as in leaves of mature trees that have completed reproductive transition. The only known phenotypic observation that differentiates between a juvenile and a reproductively mature tree in these species is the presence or floral buds/structures during the flowering season. To correlate miRNA/gene expression to maturity, we included plants from new seedlings up to mature flowering trees, with these having clear differences in their flowering competencies (in plants up to 2 years old, no floral structures were observed; on the other hand, 10-year-old avocado and macadamia and 5-year-old mango trees exhibited floral structures during the flowering season).

**Figure 1:**
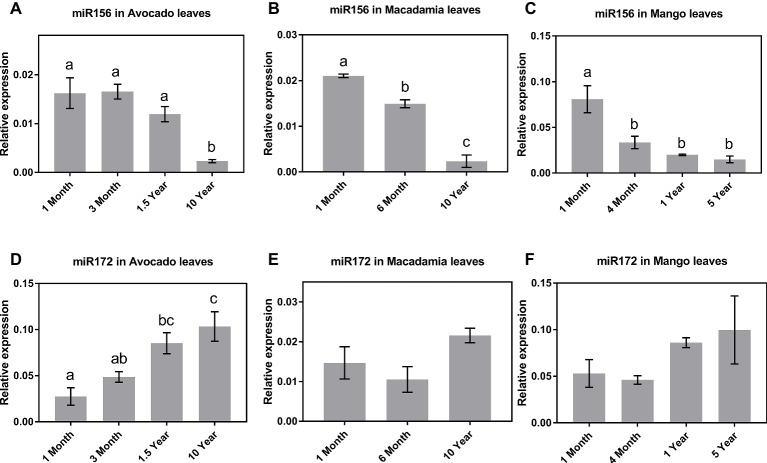
Expression of miR156 and miR172 at various stages of the avocado, mango, and macadamia life cycle. Relative abundance of miR156 and miR172 was quantified by qRT-PCR in the leaves of **(A,D)** avocado, **(B,E)** macadamia, and **(C,F)** mango. Error bars represent standard error of the mean (*n* = 3 biological pools of 6–15 plants), and significant differences calculated by one-way ANOVA are shown by different letters (*p* < 0.05).

The qRT-PCR results in all three species revealed that, of the tissues tested, miR156 is most highly expressed in leaves of young seedlings (Figures 1A–C). Specifically, in avocado seedlings, miR156 abundance started declining 18 months after germination and was lowest (*p* < 0.05) in mature (>10-year-old) trees (Figure 1A). Macadamia leaves exhibited a steady decline in miR156 abundance from juvenile to mature trees (*p* < 0.05; Figure 1B). Interestingly, miR156 abundance in mango, which was highest 1 month after seedling germination, decreased and remained significantly lower at all other age-related time points (Figure 1C; *p* < 0.05). Taken together, this may suggest that miR156 action in phase transition is conserved in horticultural tree crops but the rate of decrease in its abundance differs between species.

The observed miR172 abundance pattern was quite different among the three crop species investigated. Consistent with miR172’s proposed role in other species (5), in avocado, the lowest expression levels were detected in 1-month-old seedlings, while the highest expression was observed in 10-year-old reproductively mature trees (Figure 1D). In contrast, in macadamia and mango, miR172 expression did not significantly vary between juvenile and reproductively mature plants although the average abundance was highest in mature trees (Figures 1E,F).

### miR156 As a Negative Regulator of *SPL3/4/5*

In *Arabidopsis*, 10 members of the *SPL* gene family are targeted by miR156 (Xu et al., 2016). Of these, *SPL3/4/5* and *SPL9* have been implicated in phase transition in the leaves of the plants (Wang et al., 2011). To explore whether the variation in miR156 expression during phase transition may have a functional significance in our three selected fruit tree species, transcript homologs for *Arabidopsis SPL* genes, all possessing miR156 target sites, were identified in avocado, mango, and macadamia using in-house genomics and transcriptomic resources (Supplementary Figure S1) (see Materials and Methods section).

**Figure 2:**
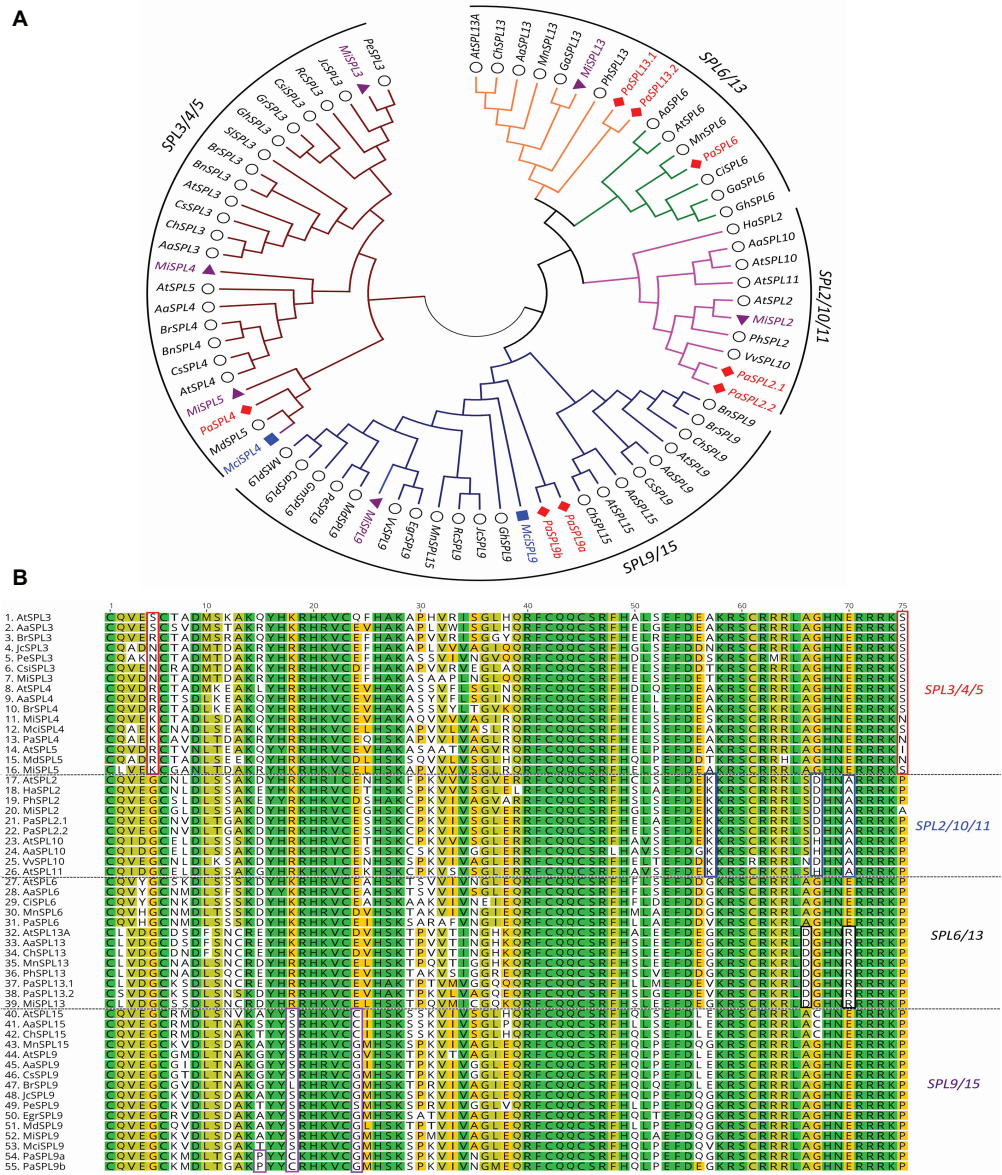
Phylogenetic analysis of miR156-targeted *SPL* genes from different plants including avocado, mango, and macadamia. **(A)** miR156-targeted *SPL* phylogenetic tree constructed with Maximum likelihood analysis of already published sequence from other crops (Additional file 1: Supplementary Table S4) with identified *SPL* transcripts from avocado, mango, and macadamia (See Materials and Methods section for tree parameters). **(B)** Sequence alignment of SBP domain of the *SPL3/4/5*, *SPL2/10/11*, *SPL6/13*, and *SPL9/15*. The unique or dissimilar sequence from each clade is shown in the box. The *SPL* transcripts from other crops (Supplementary Table S2) were aligned with the identified transcripts from the tree crops. Species codes: *Aa, Arabis alpine; At, Arabidopsis thaliana; Bn, Brassica napus; Br, Brassica rapa; Car, Cicer arietinum; Ch, Cardamine hirsute; Ci, Citrus unshiu; Csi, Citrus sinensis; Cs, Camelina sativa; Egr, Eucalyptus grandis; Ga, Gossypium arboretum; Gh, Gossypium hirsutum; Gr, Gossypium raimondii; Gm, Glycine max; Ha, Helianthus annuus; Jc, Jatropha curcas; Mci, Macadamia integrifolia; Md, Malus domestica; Mi, Mangifera indica; Mn, Morus notabilis; Mt, Medicago truncatula; Pa, Persea americana; Pe, Populus euphratica; Ph, Petunia x hybrida; Rc, Ricinus communis; Sl, Solanum lycopersicum; Vv, Vitis vinifera.*

A phylogenetic tree of miR156-targeted *SPL* genes was first constructed using full protein sequences from different species (Figure 2A). As shown in Figure 2A, avocado, macadamia, and mango *SPL* genes clustered into four broad clades: (1) *SPL3/4/5*, (2) *SPL9/SPL15*, (3) *SPL2*/*10*/*11*, and (4) *SPL6*/*SPL13* (Guo et al., 2008). The tree-crop sequences grouped in accordance with their expected relationships to the same genes in *Arabidopsis* and other plants. Furthermore, a multiple sequence alignment of the *SPLs’* functional SBP domain was constructed and further confirmed these four clades based on sequence similarities/differences (Figure 2B; Birkenbihl et al., 2005).

**Figure 3:**
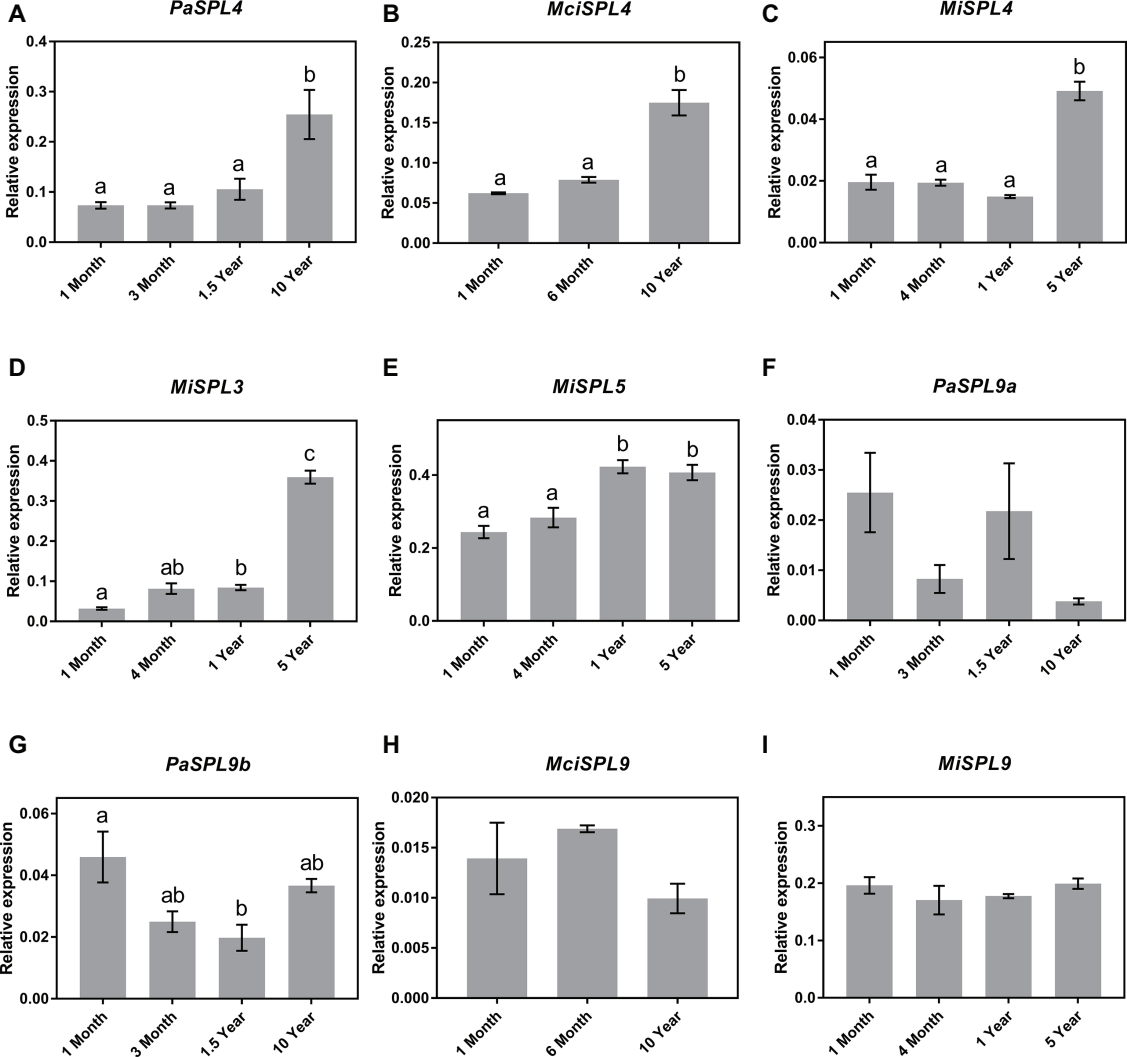
Expression of miR156 putative target genes at various stages of the avocado, mango, and macadamia life cycle. Relative abundance was quantified by qRT-PCR in leaves of miR156-targeted **(A)** *PaSPL4*, **(B)** *MciSPL4*, **(C)** *MiSPL4*, **(D)** *MiSPL3*, **(E)** *MiSPL5*, **(F)** *PaSPL9a*, **(G)** *PaSPL9b*, **(H)** *MciSPL9*, and **(I)** *MiSPL9*. Error bars represent standard error of the mean (*n* = 3 biological pools of 6–15 plants), and significant differences calculated by one-way ANOVA are shown by different letters (*p* < 0.05).

Following identification of the distinct *SPL* genes, the identified *SPL*3*/4/5* and *SPL9* transcripts were next quantified in the age-related leaf samples by qRT-PCR for correlation to miR156 abundance and developmental age. The qRT-PCR analysis revealed a significant upregulation in the transcript abundance of *SPL4* genes in the three tree crop species (*PaSPL4, MciSPL4* and *MiSPL4*) from juvenile to reproductively mature trees (Figures 3A–C). This is consistent with previous reports of miR156-*SPL3/4/5* regulatory module in phase transition (Wang et al., 2011). Only in case of mango, we were also able to identify *SPL3* and *SPL5* transcripts in available transcriptomic data. Each gene had a unique, yet similar, expression pattern to *MiSPL4*. *MiSPL3* expression was low in juvenile mango trees and started increasing during phase transition becoming highest in 5-year-old trees (Figure 3D). *MiSPL5* transcript abundance was similarly low in 1-month- and 4-month-old trees and reached a maximum level in 1-year-old trees (Figure 3E). These data may suggest that each gene plays a role during phase transition of mango and could be used as a potential marker to differentiate distinct phases.

Lastly, two *SPL9* homologs were identified in avocado (*PaSPL9a* and *PaSPL9b*), one in macadamia *(MciSPL9)*, and one in mango *(MiSPL9)*. Surprisingly, *SPL9* expression in leaves of the three crop species showed no anticorrelation with miR156 or age of the trees (Figures 3F–I). This is in contrast with previously observed expression pattern of SPL9 in other plants like *Arabidopsis, Eucalyptus*, and *Populus* where SPL9 expression anticorrelates with miR156 and is highly expressed in reproductively mature plants (Wang et al., 2009, 2011).

### *AP2-Like* and miR172: No Obvious Correlation

**Figure 4:**
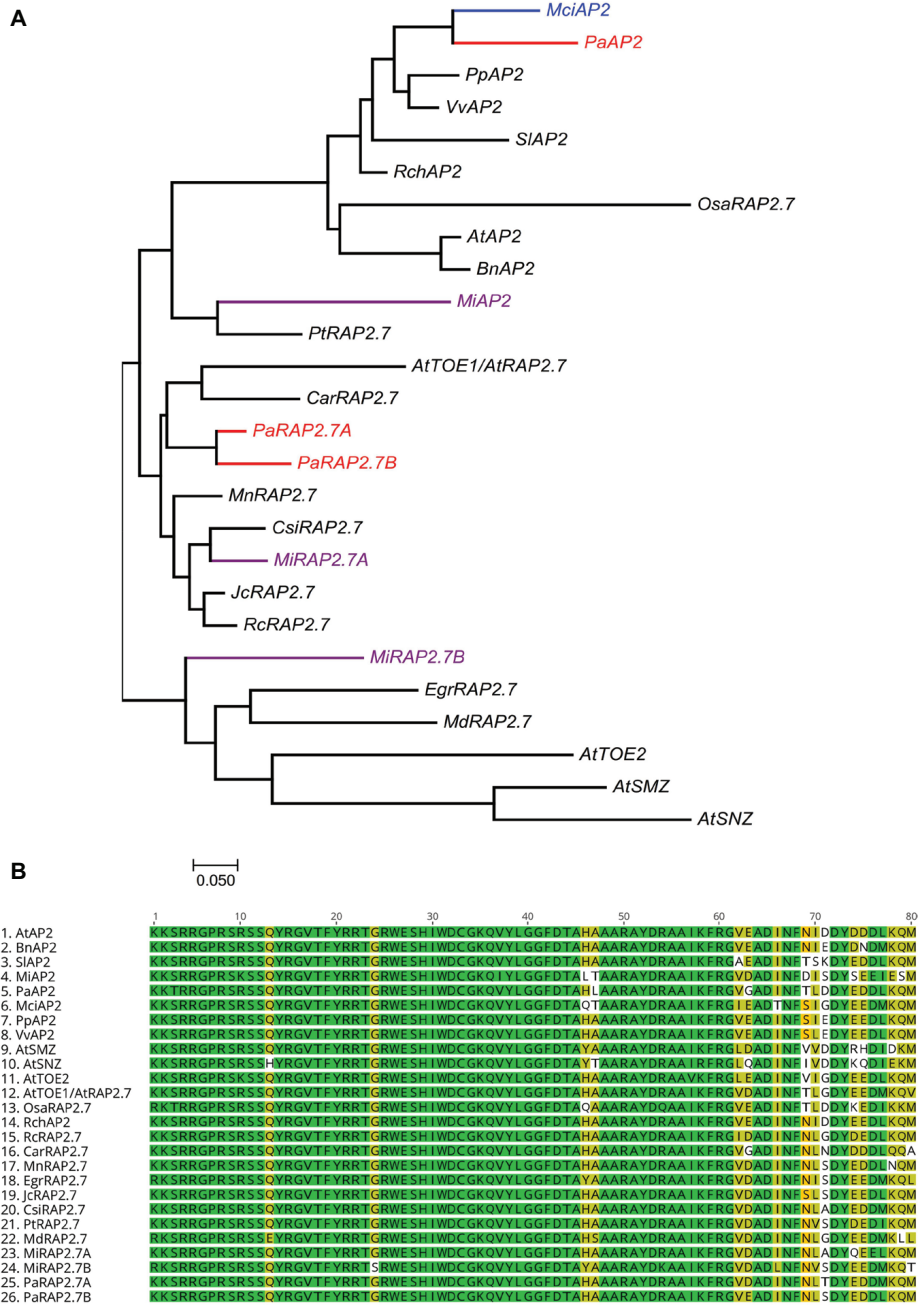
Phylogenetic analysis of miR172-targeted *AP2-like* genes. **(A)** miR172 targeted *AP2-like* gene phylogenetic tree constructed with Maximum likelihood analysis (See Material and Methods section for tree parameters), **(B)** Sequence alignment of highly conserved region (AP2 domain) of miR172 targeted *AP2*-like genes from different crops (Supplementary Table S3). Species codes: *At, Arabidopsis thaliana; Bn, Brassica napus; Car, Cicer arietinum; Csi, Citrus sinensis; Egr, Eucalyptus grandis; Jc, Jatropha curcas; Mci, Macadamia integrifolia; Md, Malus domestica; Mi, Mangifera indica; Mn, Morus notabilis; Osa, Oryza sativa; Pa, Persea americana; Pp, Prunus persica; Pt, Populus trichocarpa; Rc, Ricinus communis; Rch, Rosa chinensis; Sl, Solanum lycopersicum; Vv, Vitis vinifera.*

Homologs of miR172 target genes were also identified in the tree crops and assayed across the phase change-related samples using qRT-PCR. We identified three closely related *AP2* homologs containing miR172 target sites in avocado (*PaAP2, PaRAP2.7a (TOE1), PaRAP2.7b*) and in mango (*MiAP2, MiRAP2.7a, MiRAP2.7b*), and one in macadamia (*MciAP2*) (Supplementary Figure S2). A phylogenetic tree of miR172-targeted *AP2-like* genes was constructed using full protein sequences from different species (Figure 4A). *PaAP2* and *MciAP2* were closely related, while *MiAP2* was clustered closely with *Populus* (Figure 4A). Furthermore, a multiple sequence alignment of the highly conserved AP2 domain (~60 amino acids) was constructed which suggests its conservation in different AP2-like proteins is so high that only few bases were dissimilar (Figure 4B; Kim et al., 2006).

**Figure 5:**
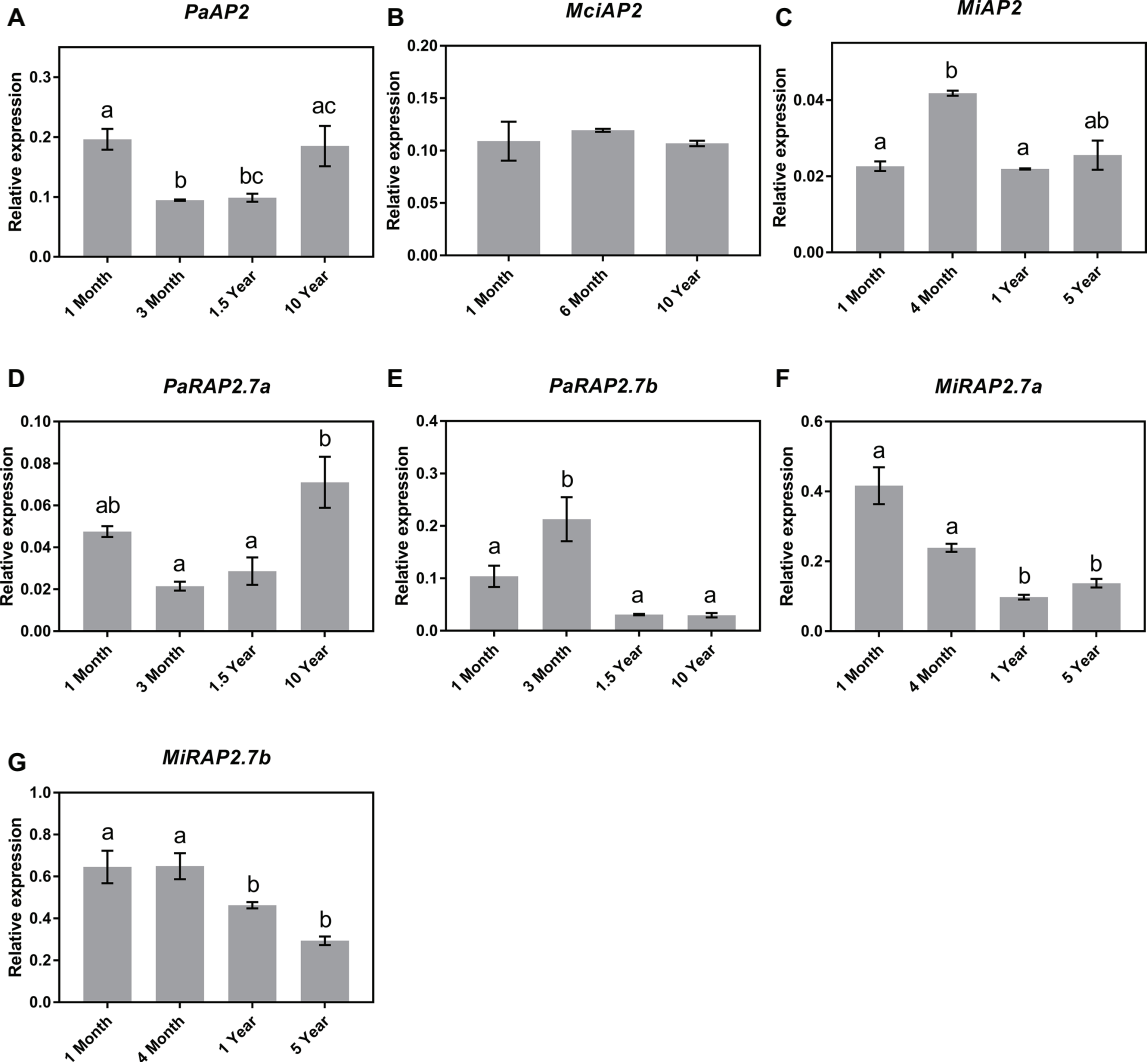
Expression of miR172 putative target genes at various stages of the avocado, mango, and macadamia life cycle. Relative expression was determined by qRT-PCR in leaves of **(A)** *PaAP2*, **(B)** *MciAP2*, **(C)** *MiAP2*, **(D)** *PaRAP2.7a*, **(E)** *PaRAP2.7b*, **(F)** *MiRAP2.7a*, and **(G)** *MiRAP2.7b*. Error bars represent standard error of the mean (*n* = 3 biological pools of 6–15 plants), and significant differences calculated by one-way ANOVA are shown by different letters (*p* < 0.05).

Next, qRT-PCR analyses showed that despite the significant increase in miR172 abundance observed across the juvenile to adult transition in avocado, the expression of the *AP2-*like genes in avocado leaves did not correspond to miR172 abundance or phase transition, as such *PaAP2* and *PaRAP2.7a* were expressed highest in both the 1-month- and 10-year-old samples (Figures 5A,D), *PaRAP2.7b* was highest in 3-month-old avocado samples and was low at all other time points (Figure 5E). This, however, does not rule out the possibility of translational inhibition by miR172 since the observed data are based on RNA transcript abundance. Likewise, *MciAP2* did not show any transcript variation pattern to the age of the trees investigated, which is also consistent with the lack of miR172 variation seen across the samples (Figures 5B,C). Furthermore, significant transcriptional changes were observed between different age samples for *MiAP2*. However, these differences simply do not follow the expected pattern. Interestingly, however, the two additional mango *AP2-like* homologs (*MiRAP2a* and *MiRAP2b*) significantly decreased in transcript abundance over the age of the tree (*p* < 0.05) (Figures 5F,G).

### miR159 as a Potential Regulator of miR156

Recently, it has been suggested that miR159 indirectly represses *miR156* transcription by targeting *MYB33* transcripts for degradation, thereby facilitating timely phase transition (Guo et al., 2017). Here, we used the mature sequence of miR159 (downloaded from miRBASE, www.mirbase.org) to validate the presence of miR159 in the small RNA sequecing data for the fruit tree crops investegated. miR159 transcript abundance was then quantified by qRT-PCR in the age-related samples of each species. No correlation was observed between miR159 expression and the age of the trees, or with miR156 transcript level in the tree crops investigated (Supplementary Figure S3). This trend was also validated through small RNA sequencing of the 1-month-old and mature/flowering samples for all three trees (Supplementary Figure S4).

### Additional miRNAs in Phase Change

To further explore the potential role of additional miRNAs in phase transition in horticultural crops, we compared small RNA sequencing data from leaves of the youngest (1-month-old) and oldest trees for each species (10-year-old trees for avocado and macadamia; 5-year-old trees for mango). Known plant miRNAs were predicted by alignment of small RNA reads to miRbase reference miRNAs (see text footnote 8) and candidates selected based on differential expression between juvenile and mature leaves of all three species (Supplementary Figure S5). To provide confidence in the small RNA data, we first confirmed that the above qRT-PCR data for miR156 and miR172 were replicated in the small RNA-seq. Accordingly, high miR156 read count in 1-month-old trees compared to 10-year-old trees (5-year-old trees for mango) in all three species while miR172 was 4-fold higher in mature trees of avocado, but less than 2-fold in mango and macadamia (Supplementary Figure S6). Of all other known microRNAs, three new potential candidates (miR164, miR394, and miR396), were also identified as being differentially regulated according to tree age in all three species (Supplementary Figure S5). However, no statistically significant difference between the expression of these miRNAs and the age of the trees was observed when validated by qRT-PCR (Supplementary Figures S7A–C). Taken together, these data suggest functionally conserved regulation of miR156 in vegetative phase transition of horticultural tree crops, and possibly across the plant kingdom. Meanwhile, a clear correlation between tree age and the expression of miR172, miR159, or other microRNAs, was not supported.

### *APETALA1*, *SPL4*, and *SOC1* in Phase Transition

**Figure 6:**
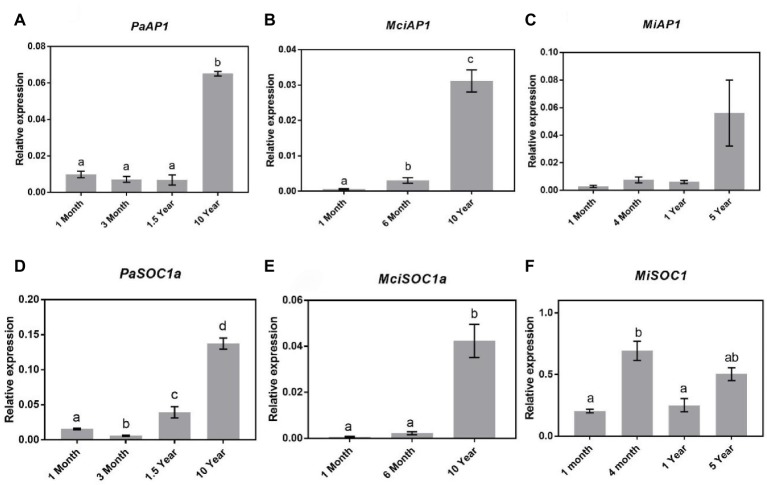
Expression of *APETALA1* and *SOC1* at various stages of the avocado, mango, and macadamia life cycle. Relative expression of *AP1* and *SOC1* was quantified by qRT-PCR in leaves of **(A)** *PaAP1*, **(B)** *MciAP1*, **(C)** *MiAP1*, **(D)** *PaSOC1a*, **(E)** *MciSOC1a*, and **(F)** *MiSCO1*. Error bars represent standard error of the mean (*n* = 3 biological pools of 6–15 plants), and significant differences calculated by one-way ANOVA are shown by different letters (*p* < 0.05).

Studies in *Arabidopsis* have shown that *SOC1* regulates *SPL*s and subsequently *AP1* expression during floral transition (Yamaguchi et al., 2009; Jung et al., 2012). It is pertinent to mention that *AP1*-encoding genes were previously cloned and their expression sites were monitored in avocado and mango (Nakagawa et al., 2012; Ziv et al., 2014). Specifically, in avocado, *AP1*-encoding gene was shown to be expressed both in leaves and in floral buds, implying that in this crop, this gene does not encode floral-specific identity genes (Ziv et al., 2014). Here, to assess the possible role of *AP1* in the phase transition in horticultural tree crops, we profiled the expression of *AP1* homologs in avocado (*PaAP1*), mango (*MiAP1*), and macadamia (*MciAP1* = identified from our transcriptomic data). The qRT-PCR expression analysis revealed a significant increase of *PaAP1*, *MiAP1*, and *MciAP1* transcript abundance in already flowering trees (Figures 6A–C). Their transcript level was minimal in young trees and was positively correlated with *SPL3/4/5*, consistent with its proposed putative role in reproductive phase transition (Murai et al., 2003; Huang et al., 2014; Chen et al., 2015a).

Given that both *SPL4* and *AP1* showed patterns of expression correlating to phase transition in tree crops investigated, we also profiled *SOC1* homologs following their identification in our available transcriptome data (*PaSOC1a, MciSOC1a*, and *MiSOC1*). Interestingly, in avocado, qRT-PCR analysis showed a steady increase in *PaSOC1a* expression after 3 months of age with significantly higher expression in 10-year-old reproductively mature trees (Figure 6D). Similarly, macadamia *SOC1a* expression also increased as the tree aged, being lowest in 1-month-old trees and significantly upregulated in mature trees (Figure 6E). On the other hand, *MiSOC1* expression was highest in 4-month-old trees and thus not correlated to phase transition, *SPL4*, or *AP1* (Figure 6F). These results might support a putative regulatory connection between *SOC1, SPL4,* and *AP1* expression in avocado and macadamia, and may suggest a possible association to maturation in these species.

### Profiling *Trehalose-6-Phosphate Synthase 1* in Juvenile and Adult Trees

A recent study has provided convincing evidence showing that *TPS1* activity in *Arabidopsis* regulates flowering in the leaves and in the SAM (Wahl et al., 2013). Here, homologs of *TPS1* in avocado, mango, and macadamia were identified using available transcriptomic/genomics data and profiled using qRT-PCR. In avocado, *PaTPS1* expression was highest during the early stages of the life cycle and was reduced after 18 months of age (Supplementary Figure S8A). A similar expression pattern was observed for *MciTPS1* where lowest expression was observed in already mature flowering trees (Supplementary Figure S8B). This is in contrast to the data for *Arabidopsis*, which revealed an increasing *TPS1* level as plants ages. Interestingly, in mango trees, no notable change in *MiTPS1* expression was seen (Supplementary Figure S8C).

## Discussion

Various endogenous factors interact with environmental cues to facilitate vegetative to reproductive phase transition in annual as well as perennial plants. However, limited information regarding the molecular regulators of phase transition is available in commercially significant horticultural tree crops, mainly due to their complex life cycle and limited genomic resources. Here, we employed a molecular approach to explore markers for juvenility and phase transition in three key horticultural tree crops: mango, avocado, and macadamia, which have significantly long juvenile phases impacting breeding and productivity. It is important to mention that there are no known phenotypic markers for vegetative phase transition in these crops during vegetative to reproductive development. One of the most common markers that is used in other plants is the leaf shape; for example, in *Eucalyptus*, juvenile leaves are morphologically different than the adult leaves (Wang et al., 2011). However, in case of avocado, mango, and macadamia, no specific morphological features were observed for leaves, or other structures, from different age samples. The only known phenotypic observation that differentiates between a juvenile and a reproductively mature tree in these species is the presence or floral buds/structures on a reproductively mature tree during the flowering season.

These crops have diverse phylogenetic origins and belong to the *Lauraceae* (avocado), *Anacardiaceae* (mango), and *Proteaceae* (macadamia) families, thought to have diverged from the model species *Arabidopsis*, more than 60 million years ago (Costello et al., 2009; Schaffer et al., 2012; Christenhusz et al., 2017). As reproductive strategy is essential for the relative success of all species during evolution, we hypothesized that conserved molecular controls for reproductive transition and flowering may provide markers for this process across commercially important crop species.

To date, miR156 and miR172 activity in leaves has been shown to act as a master regulator for the juvenile to adult phase transition in model species and some woody trees (Wang et al., 2011; Zhang et al., 2015; Feng et al., 2016; Jia et al., 2017; He et al., 2018). Here, our results revealed a conserved pattern of miR156 expression during vegetative phase transition in three diverse horticultural tree crops, suggesting that miR156 could be used as a potential juvenility marker (Figures 1A–C). We then looked for evidence of miR156-regulatory target modules in these trees by comparing the abundance of reported target genes including *SPL3/4/5* and *SPL9* with miR156 (Figure 3). Specifically, our finding showing that *MiSPL3/4/5, PaSPL4*, and *MciSPL4* exhibited low expression level in the juvenile phase, when miR156 abundance was high, and were upregulated in the reproductive phase, which suggests a potential conserved role for miR156-*SPL3/4/5* model, in regulating phase transition in these fruit tree species.

Surprisingly, however, no correlation between *SPL9* expression and either miR156 abundance or phase transition was observed in the leaves sampled from these tree crops (Figures 3F–I). This observation might suggest a lack of miR156-*SPL9* transcriptional regulatory module in leaves of these species, yet it does not rule out a possible translational or posttranslational regulation of *SPL9* protein in phase change. In this context, it is also important to note that in *Arabidopsis*, and some woody tree plants, it has been demonstrated that *SPL9* acts in phase transition downstream of miR156, partly though positively regulating miR172 (Schwarz et al., 2008; Wu et al., 2009; Wang et al., 2011). Consistent with there being no change in *MiSPL9* and *MciSPL9* expression during phase transition in mango and macadamia, we did not observe a significant change in miR172 abundance in the corresponding leaf samples. (Figures 1E,F). This may suggest a possible disconnect between miR156 and *SPL9* and miR172 in these two species but does not rule out a regulatory interaction between SPL9 protein and miR172 expression. In avocado, on the other hand, miR172 expression increased incrementally as the trees aged, in a way that anticorrelated with miR156; yet, its expression did not correspond to the accumulation of either *PaSPL9a* or *PaSPL9b* mRNAs (Figure 1D). A possible explanation of this result is that a miR156-miR172 regulatory model is active in avocado leaves during phase change, independent of *SPL9* transcriptional regulation. It remains to be determined, however, whether miR156 may show translational repression of *PaSPL9* as was shown in model organisms (Huijser and Schmid, 2011; Wang et al., 2011). Taken together, we show that *SPL9* transcript abundance in leaves is not associated with miR156 abundance or phase change in any of the tree crops examined here and, thus, it cannot be considered as a marker for phase transition. Similarly, absence of a conserved pattern of miR72 expression, during vegetative phase transition, in macadamia and mango rules out the possibility of using it as marker for maturity level. This emphasizes the need to further investigate the role of *SPL9* and miR172 in these trees, as well as other species.

Furthermore, as mentioned above, *SPL3/4/5* genes act as direct upstream activators of floral meristem identity genes including *AP1* in *Arabidopsis* (Yamaguchi et al., 2009), which by itself is positively regulated by the floral integrator *SOC1* (Jung et al., 2012). This signaling scheme, termed *SOC-SPL* module, is proposed to serve as a link that integrates external and internal signals to promote flowering in *Arabidopsis* (Jung et al., 2012). Interestingly, here, in line with this with proposed mode of action, we found that in our diverse subtropical tree species, *SOC1-SPL4-AP1* genes show a positive correlation with the reproductive phase transition (Figure 6). This is consistent with the data for *Arabidopsis* (Jung et al., 2012; Chen et al., 2015a) and wheat (Murai et al., 2003) meristems and for the first time presents this module as a possible key marker conserved in phase transition in plants, which can be easily assayed from leaves.

Moreover, the transition to reproductive maturity in trees is also carefully tied to resource availability (Wang and Wang, 2015). As such, it has been suggested that carbohydrate availability may serve as an endogenous cue that promotes phase transition in flowering plants, partially by regulating miR156-*SPL* (Wahl et al., 2013). Indeed, distinct *SPL* genes have been recently identified as potential targets of the sugar/*TPS1* signaling pathway in *Arabidopsis*, by showing that *TPS1* was negatively correlated to miR156 and positively correlated to *SPL* transcript levels (Wahl et al., 2013). In this study, we determined if a similar phenomenon could be detected in the leaves of horticultural tree species. However, in all these tree crops, no negative correlation was observed between *TPS1* transcript abundance and miR156 level (Supplementary Figure S8). Instead, *TPS1* level decreased with the age of the trees in avocado and macadamia, as did miR156, while mango showed no variation in transcript abundance. These results are in line with what was observed in a study performed in apple where no consistent variations in *TPS* activity was found between juvenile and the adult phase and suggest that increased *TPS1* expression in leaves may not be a universally reliable indicator of miR156-mediated phase transition (Jia et al., 2017).

We also examined a possible role of miR159, which has recently been suggested to indirectly regulate miR156 *via* transcript repression of the *MYB33* transcription factor (Guo et al., 2017). The results obtained showed that miR159 abundance was not correlated to the significant decrease in miR156 and phase transition in the tree crops investigated.

To conclude, here, we suggest few potential markers for juvenility/phase transition that appear to be conserved across divergent species, from the *Brassicales* through to woody trees and now the economically important tree crops: mango, macadamia, and avocado. Avocado, mango, and macadamia are ancient angiosperm plants and were known to be cultivated tree crops in distant past (Costello et al., 2009; Schaffer et al., 2012; Christenhusz et al., 2017). It is only now that we are beginning to understand the complex array of signals that regulate reproductive transition, flowering, and fruiting in these crops. We show that miR156, *SPL4*, *AP1*, and *SOC1* abundance in leaves can potentially be used as markers to predict juvenility or reproductive competence. This information may be useful to better understand bottlenecks in breeding, productivity, and propagation of these tree crops, where maturity of the propagating material can heavily influence propagation success and productivity.

## Data Availability

All datasets generated for this study are included in the manuscript and/or the Supplementary Files.

## Author Contributions

MA, AH, and NM conceived and designed the study. MA conducted all field/glasshouse and lab experiments and analyzed, interpreted, and performed statistics on experimental data. MA and AH wrote the manuscript and performed all bioinformatics analyses. MT and AP prepared small RNA libraries. MA and SF processed small RNA raw data. VI, CB, and NM provided technical feedback and edited the manuscript. All the authors approved the final manuscript.

### Conflict of Interest Statement

The authors declare that the research was conducted in the absence of any commercial or financial relationships that could be construed as a potential conflict of interest.

## Footnotes

^1^https://www.ebi.ac.uk/ena/data/view/Taxon:Macadamia%20integrifolia

^2^https://ncbi.nlm.nih.gov

^3^http://pfam.xfam.org/

^4^http://plantgrn.noble.org/psRNATarget/

^5^https://www.ncbi.nlm.nih.gov/orffinder/

^6^https://www.bioinformatics.babraham.ac.uk/projects/trim_galore/

^7^https://jgi.doe.gov/data-and-tools/bbtools/bb-tools-user-guide/bbduk-guide/

^8^http://www.mirbase.org/
