# Supplementary material for: Juvenility and Vegetative Phase Transition in Tropical/Subtropical Tree Crops
Source: Front Plant Sci. 2019 Jun 4;10:729. doi: 10.3389/fpls.2019.00729 (PMC6558100; doi:10.3389/fpls.2019.00729)
Supplement: Supplementary file 1 [file Table_1.DOCX]

Supplementary file:


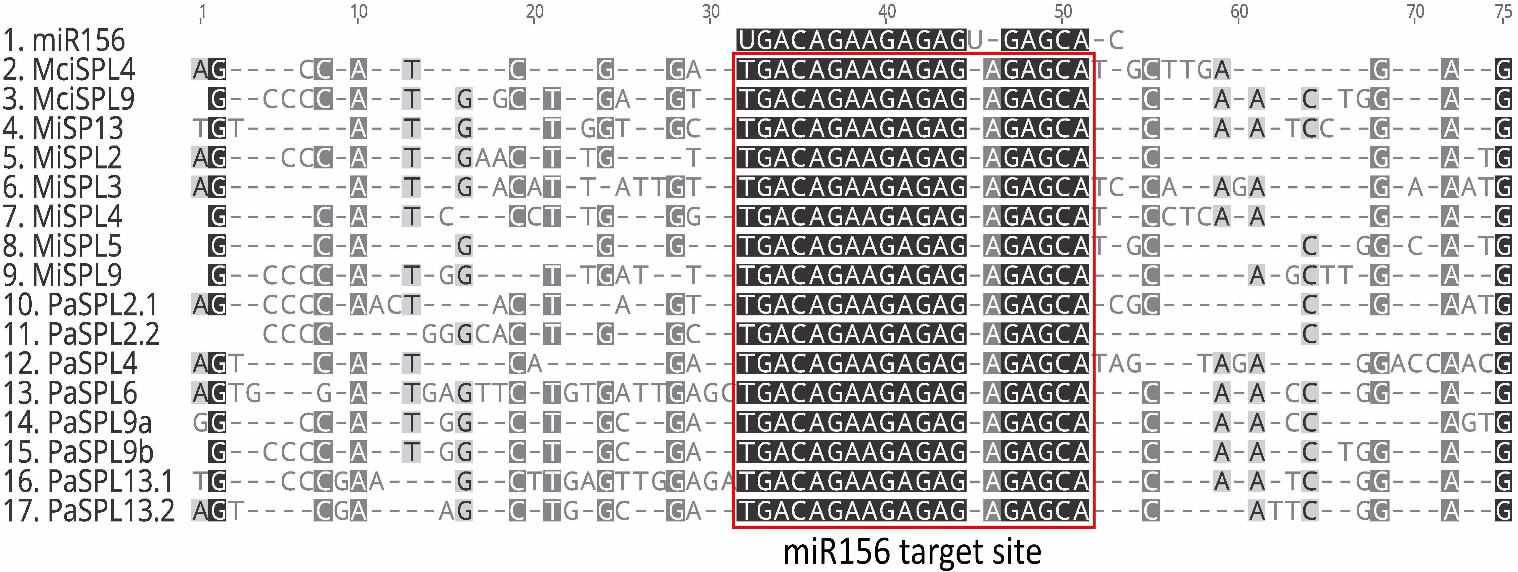


**Supplementary Figure S1.** **miR156 target site in *SPL* homologs from avocado, mango and macadamia**. All the *SPL* homologs from the horticultural tree crops were aligned using Geneious Ver 11 default setting. The region containing corresponding miR156 target site was extracted.


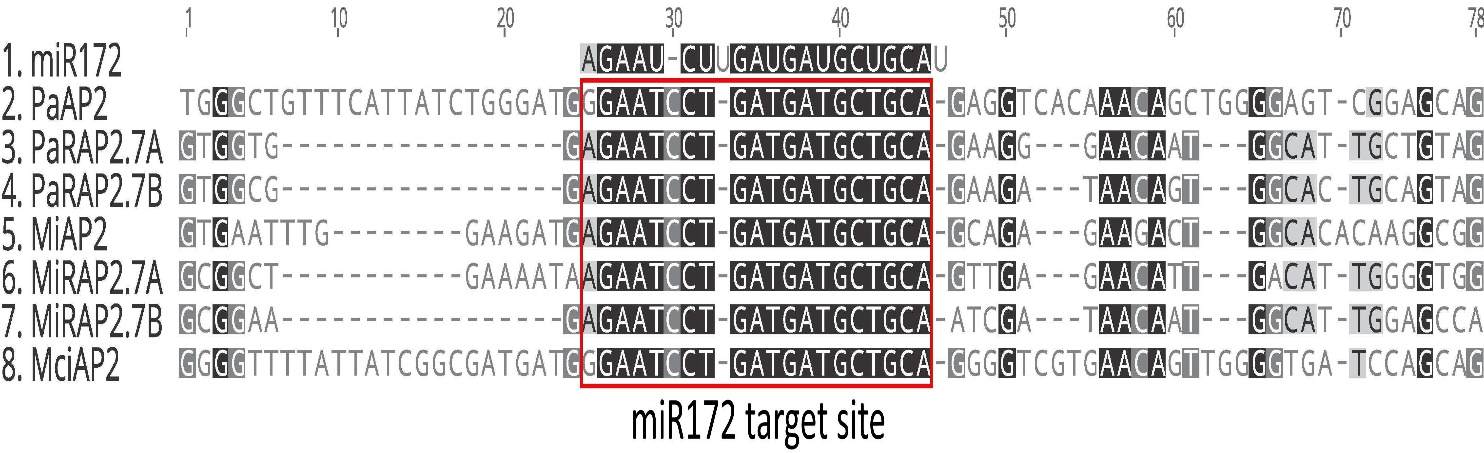
**Supplementary Figure S2.** **miR172 target site in *AP2*-like homologs from avocado, mango and macadamia**. All the *AP2-like* homologs from the horticultural tree crops were aligned using Geneious Ver 11 default setting. The region containing corresponding miR172 target site was extracted.


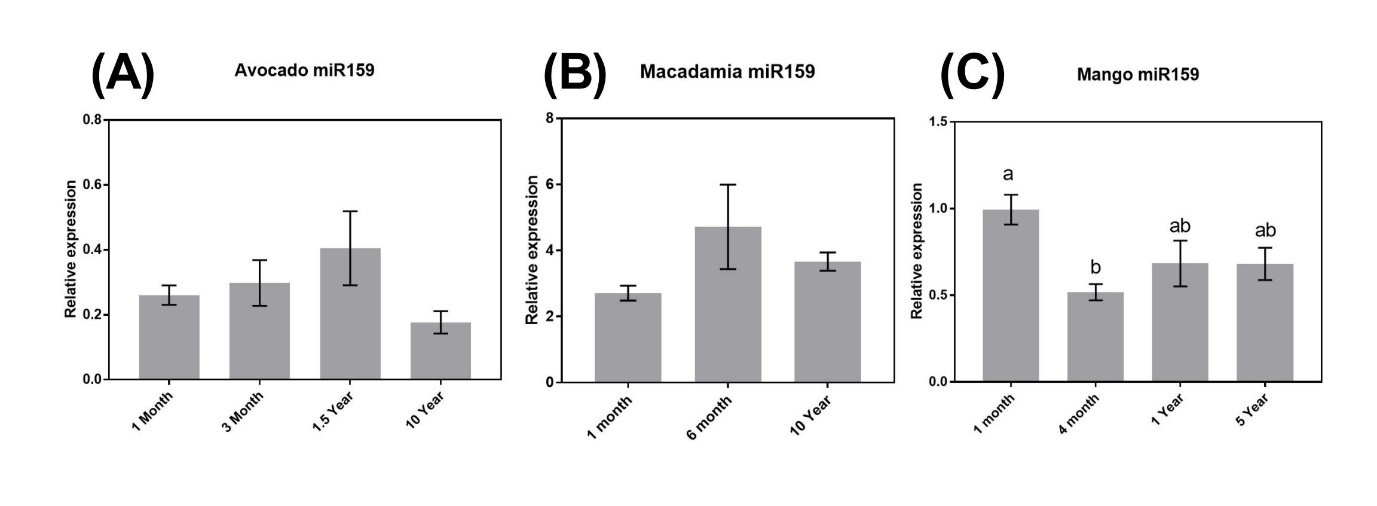


**Supplementary Figure S3. Expression of miR159 at various stages of the avocado, mango and macadamia lifecycle.** Relative expression of miR159 was quantified by qRT-PCR in leaves of, **(A)** avocado, **(B)** macadamia and **(C)** mango. Error bars represent standard error of the mean (n=3 biological pools of 6-15 plants), and significant differences calculated by one-way ANOVA are shown by different letters (p<0.05).

**Supplementary Figure S4.** **miR159 transcript read count from the small RNA sequencing data**. miR159 small RNA transcript read count from 1 month and 10 year old avocado, macadamia and mango (1 month and 5 year old) leaves.

**Supplementary Figure S5.** **Small RNA reads from 1 month and 10-year-old (5 year old mango) tree from avocado, macadamia, and mango**. Selected miRNAs transcript read count is shown in the boxes. To show relative high or low transcripts 2 colours were used, Transcript read count with Red colour show high transcripts of each miRNA from 1 month or 10 year old leaf samples from each species, respectively. Blue colour depicts low transcript reads.

**Supplementary Figure S6. Small RNA reads (miR156 and miR172) from 1 month and 10-year-old (5 year old mango) tree from avocado, macadamia, and mango leaves**.


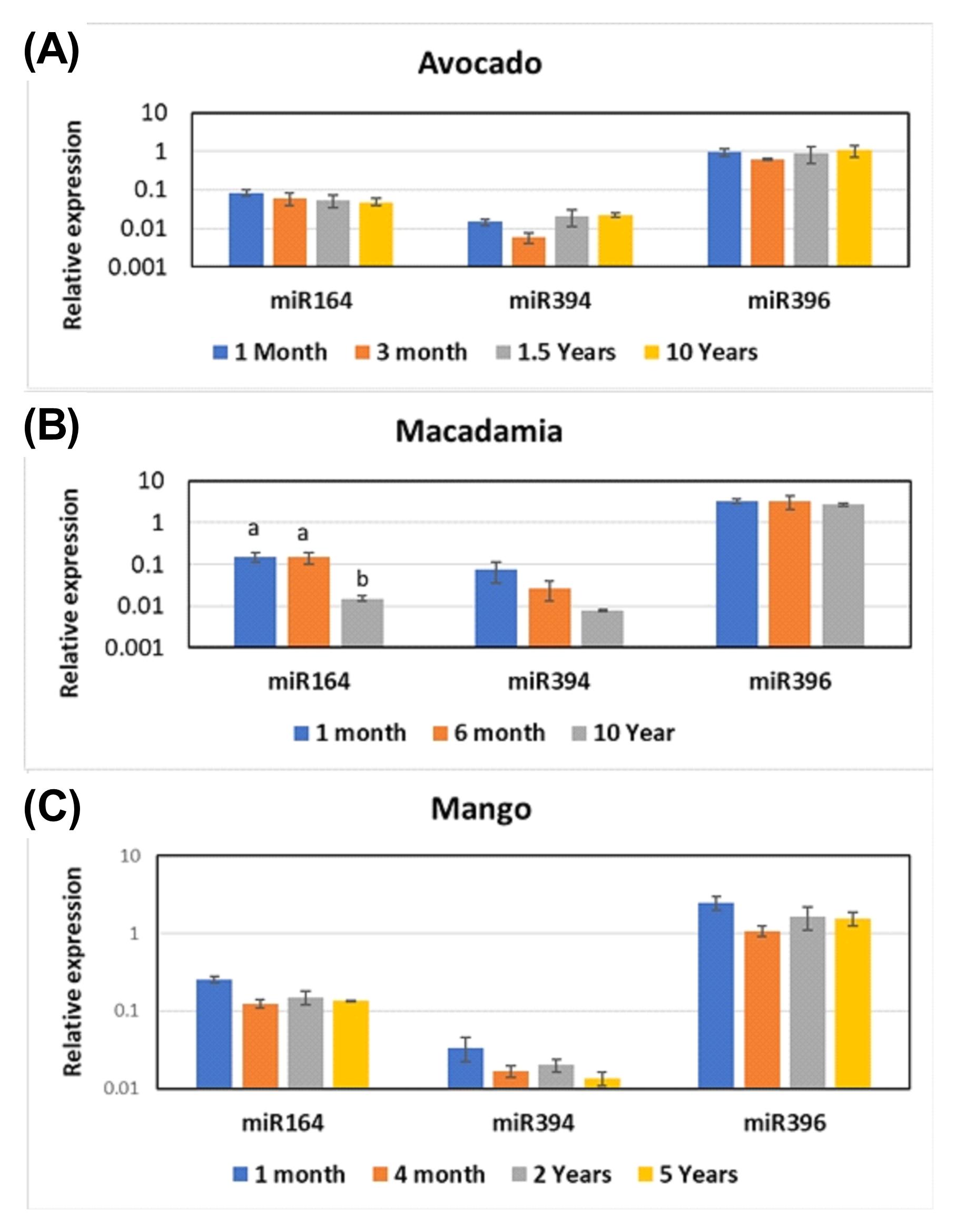


**Supplementary Figure S7.** **Additional miRNAs (miR164, miR394 and miR396) hypothesised to be involved in phase change**. Relative expression of (miR164, miR394 and miR396) in, **(A)** avocado, **(B)** macadamia and **(C)** mango. Error bars represent standard error of the mean (n=3 biological pools of 6-15 plants), and significant differences calculated by one-way ANOVA are shown by different letters (p<0.05).


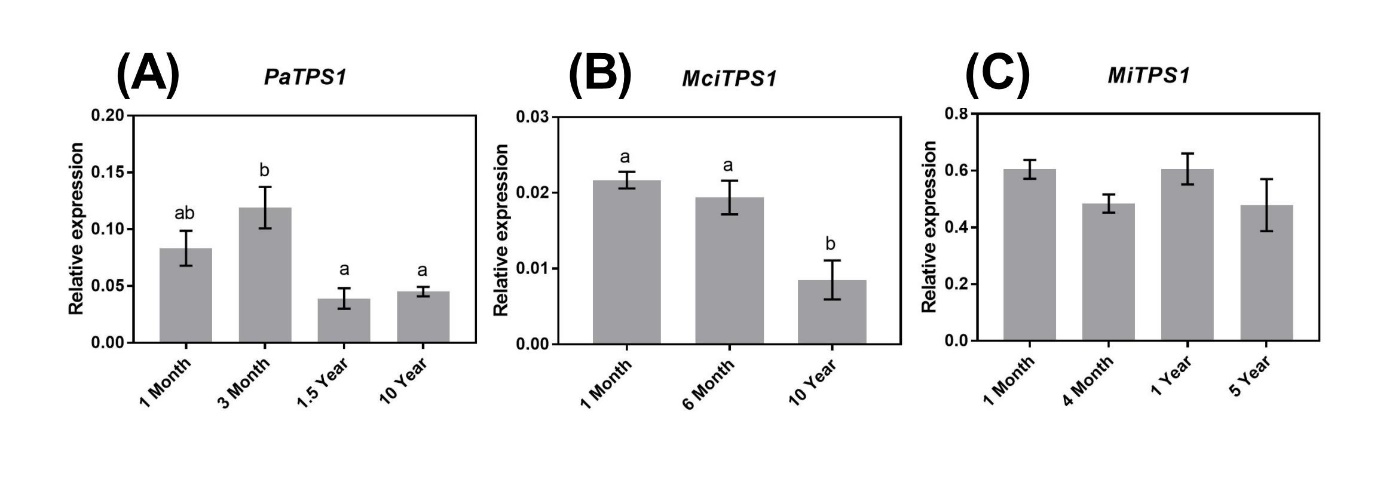


**Supplementary Figure S8. The expression of *Trehalose-6-Phosphate Synthase 1* at various stages of the avocado, mango and macadamia lifecycle.** *TPS1* Expression relative to housekeeping in, **(A)** avocado, **(B)** macadamia, and **(C)** mango. Error bars represent standard error of the mean (n=3 biological pools of 6-15 plants), and significant differences calculated by one-way ANOVA are shown by different letters (p<0.05).

**Supplementary Table S1.** New gene transcript identified from in-house transcriptome/genome data. Full length Coding region of these transcripts were predicted using Geneious software and were then submitted to NCBI repository. The integrity of each transcripts protein domain was validated using phmmr online prediction tool.

| **Gene** | **NCBI accession number** | **phmmr (pfam.xfam.org) protein domain validation results** |
| --- | --- | --- |
| *PaRAP2.7A/TOE1* | MH742966 | AP2 |
| *PaRAP2.7B* | MH742967 | AP2 |
| *PaAP2* | MH742965 | AP2 |
| *PaSPL4* | MH742962 | SBP |
| *PaSPL9a* | MH742963 | SBP |
| *PaSPL9b* | MH742964 | SBP |
| *PaSPL2.1* | MK228086 | SBP |
| *PaSPL2.2* | MK228087 | SBP |
| *PaSPL6* | MK228088 | SBP |
| *PaSPL13.1* | MK228089 | SBP |
| *PaSPL13.2* | MK228090 | SBP |
| *PaSOC1a* | MH759769 | SRF K BOX |
| *PaGAPDH* | MH759770 | GAPDH |
| *PaEF1a* | MH759771 | Elongation factor |
| *MciAP2* | MH759773 | AP2 |
| *MciSPL4* | MH759774 | SBP |
| *MciSPL9* | MH759775 | SBP |
| *MciTPS1* | MH759776 | Glycotran |
| *MciAP1* | MH759777 | SRF, K Box |
| *MciSOC1a* | MH759778 | SRF K BOX |
| *MciGAPDH* | MH759779 | GAPDH |
| *MciEF1a* | MH759780 | Elongation factor |
| *MiRAP2.7b* | MH759783 | AP2 |
| *MiRAP2.7a* | MH759784 | AP2 |
| *MiAP2* | MH759782 | AP2 |
| *MiSPL3* | MH759785 | SBP |
| *MiSPL5* | MH759787 | SBP |
| *MiSPL4* | MH759786 | SBP |
| *MiSPL9* | MH759788 | SBP |
| *MiSPL2* | MK870082 | SBP |
| *MiSPL13* | MK870083 | SBP |
| *MiAP1* | MH759790 | SRF K BOX |
| *MiSOC1* | MH759791 | SRF K BOX |
| *MiTPS1* | MH759789 | Glycotran 20 - Trehalose 6 phosphate |
| *MiEF1a* | MH759793 | EF1 |
| *MiMON1* | MH759794 | MON1 |
| *MiUBQ10* | MH759792 | UBQ10 |

**Supplementary Table S2.** *SPL* transcripts details used for alignment and phylogenetic tree.

| *Name* | *Species* | Sequenc detail |
| --- | --- | --- |
| *AaSPL3* | *Arabis alpina* | [AGP03027.1](https://www.ncbi.nlm.nih.gov/protein/AGP03027?report=genbank&log$=protalign&blast_rank=1&RID=G36U5PU2014) |
| *AaSPL4* | *Arabis alpina* | [AGP03028.1](https://www.ncbi.nlm.nih.gov/protein/AGP03028?report=genbank&log$=protalign&blast_rank=1&RID=G36U5PU2014) |
| *AaSPL6* | *Arabis alpina* | [AGP03029.1](https://www.ncbi.nlm.nih.gov/protein/AGP03029?report=genbank&log$=protalign&blast_rank=1&RID=G36U5PU2014) |
| *AaSPL9* | *Arabis alpina* | [AGP03032.1](https://www.ncbi.nlm.nih.gov/protein/AGP03032?report=genbank&log$=protalign&blast_rank=1&RID=G36U5PU2014) |
| *AaSPL10* | *Arabis alpina* | [AGP03033.1](https://www.ncbi.nlm.nih.gov/protein/AGP03033?report=genbank&log$=protalign&blast_rank=1&RID=G36U5PU2014) |
| *AaSPL13* | *Arabis alpina* | [AGP03036.1](https://www.ncbi.nlm.nih.gov/protein/AGP03036?report=genbank&log$=protalign&blast_rank=1&RID=G36U5PU2014) |
| *AaSPL15* | *Arabis alpina* | [AGP03038.1](https://www.ncbi.nlm.nih.gov/protein/AGP03038?report=genbank&log$=protalign&blast_rank=1&RID=G36U5PU2014) |
| *AtSPL2* | *Arabidopsis thaliana* | [NP_199141.1](https://www.ncbi.nlm.nih.gov/protein/NP_199141?report=genbank&log$=protalign&blast_rank=1&RID=G36U5PU2014) |
| *AtSPL3* | *Arabidopsis thaliana* | [NP_565771.1](https://www.ncbi.nlm.nih.gov/protein/NP_565771?report=genbank&log$=protalign&blast_rank=1&RID=G36U5PU2014) |
| *AtSPL4* | *Arabidopsis thaliana* | [NP_175723.1](https://www.ncbi.nlm.nih.gov/protein/NP_175723?report=genbank&log$=protalign&blast_rank=1&RID=G36U5PU2014) |
| *AtSPL5* | *Arabidopsis thaliana* | [NP_188145.1](https://www.ncbi.nlm.nih.gov/protein/NP_188145?report=genbank&log$=protalign&blast_rank=1&RID=G36U5PU2014) |
| *AtSPL6* | *Arabidopsis thaliana* | [CAB56595.1](https://www.ncbi.nlm.nih.gov/protein/CAB56595?report=genbank&log$=protalign&blast_rank=1&RID=G36U5PU2014) |
| *AtSPL9* | *Arabidopsis thaliana* | [NP_181749.1](https://www.ncbi.nlm.nih.gov/protein/NP_181749?report=genbank&log$=protalign&blast_rank=1&RID=G36U5PU2014) |
| *AtSPL10* | *Arabidopsis thaliana* | [NP_174057.2](https://www.ncbi.nlm.nih.gov/protein/NP_174057?report=genbank&log$=protalign&blast_rank=1&RID=G36U5PU2014) |
| *AtSPL11* | *Arabidopsis thaliana* | [NP_564280.1](https://www.ncbi.nlm.nih.gov/protein/NP_564280?report=genbank&log$=protalign&blast_rank=1&RID=G36U5PU2014) |
| *AtSPL13A* | *Arabidopsis thaliana* | [NP_568731.1](https://www.ncbi.nlm.nih.gov/protein/NP_568731?report=genbank&log$=protalign&blast_rank=1&RID=G36U5PU2014) |
| *AtSPL15* | *Arabidopsis thaliana* | [NP_191351.1](https://www.ncbi.nlm.nih.gov/protein/NP_191351?report=genbank&log$=protalign&blast_rank=2&RID=G36U5PU2014) |
| *BnSPL3* | *Brassica napus* | [XP_013734250.1](https://www.ncbi.nlm.nih.gov/protein/XP_013734250?report=genbank&log$=protalign&blast_rank=1&RID=G36U5PU2014) |
| *BnSPL4* | *Brassica napus* | [XP_013752440.1](https://www.ncbi.nlm.nih.gov/protein/XP_013752440?report=genbank&log$=protalign&blast_rank=4&RID=G36U5PU2014) |
| *BnSPL9* | *Brassica napus* | [XP_013687893.1](https://www.ncbi.nlm.nih.gov/protein/XP_013687893?report=genbank&log$=protalign&blast_rank=1&RID=G36U5PU2014) |
| *BrSPL3* | *Brassica rapa* | [XP_009132954.1](https://www.ncbi.nlm.nih.gov/protein/XP_009132954?report=genbank&log$=protalign&blast_rank=1&RID=G36U5PU2014) |
| *BrSPL4* | *Brassica rapa* | [XP_009147624.1](https://www.ncbi.nlm.nih.gov/protein/XP_009147624?report=genbank&log$=protalign&blast_rank=1&RID=G36U5PU2014) |
| *BrSPL9* | *Brassica rapa* | [XP_009142046.1](https://www.ncbi.nlm.nih.gov/protein/XP_009142046?report=genbank&log$=protalign&blast_rank=1&RID=G36U5PU2014) |
| *CarSPL9* | *Cicer arietinum* | [XP_004493567.1](https://www.ncbi.nlm.nih.gov/protein/XP_004493567?report=genbank&log$=protalign&blast_rank=1&RID=G36U5PU2014) |
| *ChSPL3* | *Cardamine hirsuta* | [AKC05616.1](https://www.ncbi.nlm.nih.gov/protein/AKC05616?report=genbank&log$=protalign&blast_rank=1&RID=G36U5PU2014) |
| *ChSPL9* | *Cardamine hirsuta* | [AKC05617.1](https://www.ncbi.nlm.nih.gov/protein/AKC05617?report=genbank&log$=protalign&blast_rank=1&RID=G36U5PU2014) |
| *ChSPL13* | *Cardamine hirsuta* | [AKC05619.1](https://www.ncbi.nlm.nih.gov/protein/AKC05619?report=genbank&log$=protalign&blast_rank=1&RID=G36U5PU2014) |
| *ChSPL15* | *Cardamine hirsuta* | [AKC05620.1](https://www.ncbi.nlm.nih.gov/protein/AKC05620?report=genbank&log$=protalign&blast_rank=1&RID=G38E6G38015) |
| *CiSPL6* | *Citrus unshiu* | [GAY64853.1](https://www.ncbi.nlm.nih.gov/protein/GAY64853?report=genbank&log$=protalign&blast_rank=1&RID=G38E6G38015) |
| *CsiSPL3* | *Citrus sinensis* | [XP_006467766.2](https://www.ncbi.nlm.nih.gov/protein/XP_006467766?report=genbank&log$=protalign&blast_rank=1&RID=G38E6G38015) |
| *CsSPL3* | *Camelina sativa* | [XP_010509826.1](https://www.ncbi.nlm.nih.gov/protein/XP_010509826?report=genbank&log$=protalign&blast_rank=1&RID=G38E6G38015) |
| *CsSPL4* | *Camelina sativa* | [XP_010479860.1](https://www.ncbi.nlm.nih.gov/protein/XP_010479860?report=genbank&log$=protalign&blast_rank=1&RID=G38E6G38015) |
| *CsSPL9* | *Camelina sativa* | [XP_010508605.1](https://www.ncbi.nlm.nih.gov/protein/XP_010508605?report=genbank&log$=protalign&blast_rank=1&RID=G38E6G38015) |
| *EgrSPL9* | *Eucalyptus grandis* | [XP_010057993.1](https://www.ncbi.nlm.nih.gov/protein/XP_010057993?report=genbank&log$=protalign&blast_rank=1&RID=G38E6G38015) |
| *GaSPL6* | *Gossypium arboreum* | [KHG15102.1](https://www.ncbi.nlm.nih.gov/protein/KHG15102?report=genbank&log$=protalign&blast_rank=1&RID=G38E6G38015) |
| *GaSPL13* | *Gossypium arboreum* | [XP_017635398.1](https://www.ncbi.nlm.nih.gov/protein/XP_017635398?report=genbank&log$=protalign&blast_rank=1&RID=G38E6G38015) |
| *GhSPL3* | *Gossypium hirsutum* | [XP_016730055.1](https://www.ncbi.nlm.nih.gov/protein/XP_016730055?report=genbank&log$=protalign&blast_rank=1&RID=G38E6G38015) |
| *GhSPL6* | *Gossypium hirsutum* | [NP_001314491.1](https://www.ncbi.nlm.nih.gov/protein/NP_001314491?report=genbank&log$=protalign&blast_rank=1&RID=G38E6G38015) |
| *GhSPL9* | *Gossypium hirsutum* | [XP_016742706.1](https://www.ncbi.nlm.nih.gov/protein/XP_016742706?report=genbank&log$=protalign&blast_rank=1&RID=G38E6G38015) |
| *GrSPL3* | *Gossypium raimondii* | [XP_012455582.1](https://www.ncbi.nlm.nih.gov/protein/XP_012455582?report=genbank&log$=protalign&blast_rank=3&RID=G38E6G38015) |
| *GmSPL9* | *Glycine max* | [XP_003520534.1](https://www.ncbi.nlm.nih.gov/protein/XP_003520534?report=genbank&log$=protalign&blast_rank=1&RID=G38E6G38015) |
| *HaSPL2* | *Helianthus annuus* | [OTF88071.1](https://www.ncbi.nlm.nih.gov/protein/OTF88071?report=genbank&log$=protalign&blast_rank=1&RID=G38E6G38015) |
| *JcSPL9* | *Jatropha curcas* | [XP_012087743.1](https://www.ncbi.nlm.nih.gov/protein/XP_012087743?report=genbank&log$=protalign&blast_rank=1&RID=G3984JD9015) |
| *MdSPL5* | *Malus domestica* | [ADL36827.1](https://www.ncbi.nlm.nih.gov/protein/ADL36827?report=genbank&log$=protalign&blast_rank=1&RID=G3984JD9015) |
| *MdSPL9* | *Malus domestica* | [XP_008392088.1](https://www.ncbi.nlm.nih.gov/protein/XP_008392088?report=genbank&log$=protalign&blast_rank=1&RID=G3984JD9015) |
| *MnSPL6* | *Morus notabilis* | [XP_010091609.1](https://www.ncbi.nlm.nih.gov/protein/XP_010091609?report=genbank&log$=protalign&blast_rank=1&RID=G3984JD9015) |
| *MnSPL13* | *Morus notabilis* | [XP_010086977.1](https://www.ncbi.nlm.nih.gov/protein/XP_010086977?report=genbank&log$=protalign&blast_rank=1&RID=G3984JD9015) |
| *MnSPL15* | *Morus notabilis* | [EXB93646.1](https://www.ncbi.nlm.nih.gov/protein/EXB93646?report=genbank&log$=protalign&blast_rank=1&RID=G3984JD9015) |
| *MtSPL9* | *Medicago truncatula* | [XP_003625236.2](https://www.ncbi.nlm.nih.gov/protein/XP_003625236?report=genbank&log$=protalign&blast_rank=2&RID=G3984JD9015) |
| *PeSPL3* | *Populus euphratica* | [XP_011027797.1](https://www.ncbi.nlm.nih.gov/protein/XP_011027797?report=genbank&log$=protalign&blast_rank=1&RID=G39NA1NC014) |
| *PeSPL9* | *Populus euphratica* | [XP_011033246.1](https://www.ncbi.nlm.nih.gov/protein/XP_011033246?report=genbank&log$=protalign&blast_rank=1&RID=G39NA1NC014) |
| *PhSPL2* | *Petunia x hybrida* | [AUW52967.1](https://www.ncbi.nlm.nih.gov/protein/AUW52967?report=genbank&log$=protalign&blast_rank=1&RID=G39NA1NC014) |
| *PhSPL13* | *Petunia x hybrida* | [AUW52987.1](https://www.ncbi.nlm.nih.gov/protein/AUW52987?report=genbank&log$=protalign&blast_rank=1&RID=G39NA1NC014) |
| *RcSPL3* | *Ricinus communis* | [XP_002509450.1](https://www.ncbi.nlm.nih.gov/protein/XP_002509450?report=genbank&log$=protalign&blast_rank=1&RID=G39NA1NC014) |
| *RcSPL9* | *Ricinus communis* | [XP_015582800.1](https://www.ncbi.nlm.nih.gov/protein/XP_015582800?report=genbank&log$=protalign&blast_rank=1&RID=G39NA1NC014) |
| *SlSPL3* | *Solanum lycopersicum* | [NP_001307609.1](https://www.ncbi.nlm.nih.gov/protein/NP_001307609?report=genbank&log$=protalign&blast_rank=1&RID=G39NA1NC014) |
| *VvSPL9* | *Vitis vinifera* | [NP_001267898.1](https://www.ncbi.nlm.nih.gov/protein/NP_001267898?report=genbank&log$=protalign&blast_rank=1&RID=G39NA1NC014) |
| *VvSPL10* | *Vitis vinifera* | [NP_001268193.1](https://www.ncbi.nlm.nih.gov/protein/NP_001268193?report=genbank&log$=protalign&blast_rank=1&RID=G39NA1NC014) |

**Supplementary Table S3.** miR172 targeted *AP2 like* transcripts details used for alignment and phylogenetic tree.

| Name | Species | Sequenc detail |
| --- | --- | --- |
| *RchAP2* | *Rosa chinensis* | [XP_024182693.1](https://www.ncbi.nlm.nih.gov/protein/XP_024182693.1) |
| *SlAP2* | *Solanum lycopersicum* | [NP_001234452.1](https://www.ncbi.nlm.nih.gov/protein/NP_001234452.1) |
| *VvAP2* | *Vitis vinifera* | [XP_010652782.1](https://www.ncbi.nlm.nih.gov/protein/XP_010652782.1) |
| *BnAP2* | *Brassica napus* | [XP_013707660.1](https://www.ncbi.nlm.nih.gov/protein/XP_013707660.1) |
| *AtAP2* | *Arabidopsis thaliana* | [AT4G36920.1](https://www.arabidopsis.org/servlets/TairObject?type=gene&id=127955) |
| *AtTOE1/AtRAP2.7* | *Arabidopsis thaliana* | [AT2G28550.3](https://www.arabidopsis.org/servlets/TairObject?type=gene&id=1000687991) |
| *AtTOE2* | *Arabidopsis thaliana* | [AT5G60120.2](https://www.arabidopsis.org/servlets/TairObject?type=gene&id=1000689281) |
| *AtSMZ* | *Arabidopsis thaliana* | [AT3G54990.1](https://www.arabidopsis.org/servlets/TairObject?type=gene&id=435363) |
| *AtSNZ* | *Arabidopsis thaliana* | [AT2G39250.1](https://www.arabidopsis.org/servlets/TairObject?type=gene&id=35283) |
| *CarRAP2.7* | *Cicer arietinum* | [XP_004506004.1](https://www.ncbi.nlm.nih.gov/protein/XP_004506004.1?report=genbank&log$=protalign&blast_rank=1&RID=MBUBU72H01R) |
| *CsiRAP2.7* | *Citrus sinensis* | [XP_006488203.1](https://www.ncbi.nlm.nih.gov/protein/XP_006488203.1?report=genbank&log$=protalign&blast_rank=1&RID=MBUBU72H01R) |
| *EgrRAP2.7* | *Eucalyptus grandis* | [XP_010067252.1](https://www.ncbi.nlm.nih.gov/protein/XP_010067252.1?report=genbank&log$=protalign&blast_rank=1&RID=MBUBU72H01R) |
| *JcRAP2.7* | *Jatropha curcas* | [XP_012064916.1](https://www.ncbi.nlm.nih.gov/protein/XP_012064916.1?report=genbank&log$=protalign&blast_rank=1&RID=MBUBU72H01R) |
| *MdRAP2.7* | *Malus domestica* | [XP_008369496.1](https://www.ncbi.nlm.nih.gov/protein/XP_008369496.1?report=genbank&log$=protalign&blast_rank=1&RID=MBUBU72H01R) |
| *MnRAp2.7* | *Morus notabilis* | [XP_024031787.1](https://www.ncbi.nlm.nih.gov/protein/XP_024031787.1?report=genbank&log$=protalign&blast_rank=1&RID=MBVN1MM401R) |
| *OsaRAP2.7* | *Oryza sativa* | [XP_015642711.1](https://www.ncbi.nlm.nih.gov/protein/XP_015642711.1?report=genbank&log$=protalign&blast_rank=1&RID=MBVN1MM401R) |
| *PpAP2* | *Prunus persica* | [XP_007208004.1](https://www.ncbi.nlm.nih.gov/protein/XP_007208004.1?report=genbank&log$=protalign&blast_rank=1&RID=MBVN1MM401R) |
| *PtRAP2.7* | *Populus trichocarpa* | [XP_002322849.2](https://www.ncbi.nlm.nih.gov/protein/XP_002322849.2?report=genbank&log$=protalign&blast_rank=1&RID=MBVN1MM401R) |
| *RcRAP2.7* | Ricinus communis | [XP_015581507.1](https://www.ncbi.nlm.nih.gov/protein/XP_015581507.1?report=genbank&log$=protalign&blast_rank=1&RID=MBVN1MM401R) |

**Supplementary Table S4.** Primers used for miRNA quantification.

| **No.** | **Gene** | **F Primer** | **R Primer** |
| --- | --- | --- | --- |
| 1 | miR156 | TGACAGAAGAGAGTGAGCAC | Universal Primer |
| 2 | miR172 | AGAATCTTGATGATGCTGCAT | Universal Primer |
| 3 | miR159 | TTTGGATTGAAGGGAGCTCTA | Universal Primer |
| 4 | miR164 | TGGAGAAGCAGGGCACGTGCA | Universal Primer |
| 6 | miR394 | TTGGCATTCTGTCCACCTCC | Universal Primer |
| 7 | miR396 | TTCCACAGCTTTCTTGAACTG | Universal Primer |
| 9 | U6 SnoRNA (Avo + Mango) | GGATGACACGCACAAATCGAG | Universal Primer |
| 10 | U6 SnoRNA (Macadamia) | GGATGACATGCACAAATCGAG | Universal Primer |
| 11 | 5.8S ribosomal RNA rRNA | GAATTGCAGAATCCCGTGAACC | Universal Primer |

**Supplementary Table S5.** Primers used for gene expression analysis.

| **No.** | **Gene** | **F Primer** | **R Primer** |
| --- | --- | --- | --- |
| 1 | *PaRAP2.7A/TOE1A* | GCAGTAGAGAGGGCTGAACTC | GTAACAGTAGGTAGGAAAATGGCC |
| 2 | *PaRAP2.7B* | CAACTACGAGGCTCTACTGC | GCTTTCAGCTCTGGTAATAGTAGG |
| 3 | *PaAP2* | GGAGCAGTAGTAATTGGGCTGC | CATAATCAAGGGTCAGGTAGGTC |
| 4 | *PaSPL4* | CGTTGGTTCTCTACTATGCTCTC | GTACCTACTGGCACAGATAGC |
| 5 | *PaSPL9a* | CTTTGGCAGGGTAGCATAGAG | GCTGTGATACCCGAAGCTCG |
| 6 | *PaSPL9b* | GCAGGAATCTCCAACTCCAG | TGGCACTATGACTTGGTGGA |
| 7 | *PaTPS1* | CTTCAAGCGAGGGATATGCT | TGCTGCACCCTTTGTAACAC |
| 8 | *PaAP1* | CTCAAGCAGTGTGGGGGTAG | GAAGCATCCAAGGTGGGACA |
| 9 | *PaSOC1a* | GCGCTCATTGTTTTCTCTCC | TGCTGCTTACGTCTTTGGTG |
| 10 | *PaGAPDH* | TGGGAAACTTACAGGAATGG | GTCACCCACAAAGTCAGTAGAA |
| 11 | *PaEF1a* | ATCAAGCGTGGGTTTGTTGC | TACCCGTTGCCAATCTGACC |
| 13 | *MciAP2* | CTGCTGGATCACCCCAACTG | ATGTCAGGTGGGTCTCATG |
| 14 | *MciSPL4* | ACCATTGCAGGCAGGCTGAC | GTACCAGCATGAAAGGAAGC |
| 15 | *MciSPL9* | CCTCCAGTTGAATGCTTTGC | GTTGTCCAGTTCAAGACCG |
| 16 | *MciTPS1* | ATCGCTTAGCAACCACTCGT | AATCATGGCACCAAACAACA |
| 17 | *MciAP1* | GAGGCGGATCGATAACAAGA | CGGTGGAGTACTCGAAGAGC |
| 18 | *MciSOC1a* | GCAACATTAGGGGAAGGAAGAC | GCCTTCTCTCGTAGTATTCTG |
| 20 | *MciGAPDH* | GGAGTCAGAGGGTAGCCTGA | ACCAGGAGACAAGCTTCACG |
| 21 | *MciEF1a* | GAACGTCGCAGTTAAGGATC | GAGGTGTGGCAATCAAGCAC |
| 23 | *MiRAP2.7b* | CAAACTGGGCATGGCAAACG | GAAGAAGTGCTGGTAGCG |
| 24 | *MiRAP2.7a* | CAACCGCATGGTCAGGTGAG | GTGAAACGGAGATTGAGG |
| 25 | *MiAP2* | ACAGGAAAGAGTGGCAGAG | GAAGGCACTGTGAATTTGG |
| 26 | *MiSPL3* | TCTCTTGGATGCTCTCTC | GGAAGTTTAGCCAAATATGC |
| 27 | *MiSPL5* | GCTAGAGGCAAAGTTCTGATGC | CGTACACAGCTTAGAAACAACTGC |
| 28 | *MiSPL4* | GCTCTTGAGGATGCTCTCTC | GTTCCGATTCCAATGGCTTG |
| 29 | *MiSPL9* | GATTGCTTCACAGGAGTTGC | GCATCAAGTCACTCACTCC |
| 30 | *MiAP1* | F1: ATCCTGGTCCTGAATCAACG | TAGTGCCTTTGGCTCCTTTG |
| 31 | *MiSOC1* | CTGTTGGGAGAAGGTCTGGA | TCTAGCGCGAATGTTGCTTA |
| 32 | *MiTPS1* | TGTTGCTGCGTTTCCTATTG | GGCCAGCAAATGTCTCTTTC |
| 33 | *MiEF1a* | AAGAGGCCATCAGACAAGCC | CCGGTTTCAACACGACCAAC |
| 34 | *MiGAPDH* | ATGCCAAGGCTGGTATTGCT | TCACGCGTGAACTGTATCCC |
| 35 | *MiMON1* | GACTTGCAACCGGGTTTGTC | CACCATGAAAAGGGGCTTGC |
| 36 | *MiUBQ10* | ACCCTGCATCTTGTCCTTCG | CACACACAAGCCTACCGGAT |
